# Gut microbiota’s role in the enhancement of type 2 diabetes treatment by a traditional Chinese herbal formula compared to metformin

**DOI:** 10.1128/spectrum.02412-24

**Published:** 2025-03-31

**Authors:** Chengdong Xia, Liya Yue, Yinyu Wang, Cuidan Li, Guannan Ma, Yingjiao Ju, Peihan Wang, Jie Wang, Xiaoyuan Jiang, Xiaotong Wang, Fei Chen

**Affiliations:** 1 Department of Endocrinology, Xiyuan Hospital of China Academy of Chinese Medical Sciences 425205 , Beijing, China; 2 China National Center for Bioinformation 74696 , Beijing, China; 3 Beijing Institute of Genomics, Chinese Academy of Sciences 74696 , Beijing, China; 4 University of Chinese Academy of Sciences 74519, Beijing, China; 5 State Key Laboratory of Pathogenesis, Prevention and Treatment of High Incidence Diseases in Central Asia, Clinical Medicine Institute, The First Affiliated Hospital of Xinjiang Medical University 159427, Urumqi, China; 6 Key Laboratory of Viral Pathogenesis & Infection Prevention and Control (Jinan University), Ministry of Education, Guangzhou, China; 7 Beijing Key Laboratory of Genome and Precision Medicine Technologies, Beijing, China; University of Nebraska-Lincoln, Lincoln, Nebraska, USA

**Keywords:** traditional Chinese medicine, type 2 diabetes mellitus, gut microbiota, metformin

## Abstract

Type 2 diabetes mellitus (T2DM) is a rapidly increasing metabolic disorder that poses a significant threat to global public health. Recent evidence suggests that targeting the gut microbiota through dietary and pharmaceutical interventions can effectively manage T2DM. In this study, we developed a novel Chinese herbal formula, CCM, specifically for T2DM, composed of *Coptis rhizoma*, *Cinnamomi cortex*, and *Mume fructus*. To evaluate CCM’s efficacy and explore its underlying mechanisms, particularly the role of the gut microbiota, diabetic C57/db/db mice were administered different doses of CCM (low, medium, high) for 4 weeks, with normal C57 mice as healthy controls and metformin as a positive control. Comprehensive clinical indicators of T2DM were measured before and after treatment. High-throughput sequencing was used to assess changes in gut microbiome composition and function. Our results showed that CCM treatment, especially at medium and high doses, resulted in more significant improvements in blood glucose, lipid profiles, and body weight compared to metformin. The CCM-treated group also exhibited more significant changes in the microbial community structure compared to the metformin group, notably enriching three beneficial microbes (>40%): *Bacteroidetes* spp., *Akkermansia* spp., and *Parabacteroides* spp., which correlated with improved diabetic parameters. Further analysis identified that all four microbial metabolic pathways linked to lowering blood glucose were exclusively enriched in the CCM-treated group. Of the 10 pathways related to improved blood lipid levels, five were unique to CCM. These unique pathways enriched by CCM may explain its superior therapeutic effects, indicating its distinct mechanisms in modulating gut microbiota.

**IMPORTANCE:**

Our study demonstrates that CCM outperforms metformin in managing key clinical indicators in type 2 diabetes mellitus (T2DM) model mice and induces more significant alterations in gut microbiota composition and function. Notably, the uniquely enriched beneficial microbes and microbial metabolic pathways in the CCM samples may explain its enhanced therapeutic effects compared to metformin. Consequently, these findings suggest that CCM offers a promising therapeutic strategy for T2DM, and further provide valuable insights into potential probiotic candidates (such as *Bacteroidetes* spp., *Akkermansia* spp., and *Parabacteroides* spp.) and newly identified functional pathways (such as chondroitin sulfate degradation, geraniol degradation, biotin biosynthesis, colonic acid building blocks biosynthesis, and the biosynthesis of vancomycin group antibiotics) that could be targeted for therapeutic intervention.

## INTRODUCTION

Type 2 diabetes mellitus (T2DM) is a rapidly increasing metabolic disorder that poses a significant public health challenge globally (1). It primarily arises from insulin resistance, often induced by high-calorie diets and other unhealthy lifestyle habits (2). Recently, the rapid development of metagenomics has highlighted the gut microbiota’s critical role in the pathophysiology of T2DM (3–5). For instance, a metagenome-wide association study identified a gut microbial dysbiosis in T2DM patients, notably a significant reduction in butyrate-producing bacteria (6). Moreover, fecal microbiota transplantation from individuals with T2DM or obesity into germ-free mice has been shown to have the ability to induce T2DM phenotypes, indicating that gut microbes can causatively influence metabolic diseases such as T2DM and obesity (7, 8).

Further clinical trials have demonstrated that increasing dietary fiber intake can mitigate T2DM symptoms by modulating the gut microbiota (9). Recent studies suggest that dietary fibers improve glucose metabolism and reduce insulin resistance by altering the gut microbiota (10). Specifically, high-fiber diets such as inulin-type fructans have been shown to alleviate T2DM symptoms by modifying the gut microbial community (11).

The above findings emphasize the potential of targeting the gut microbiota through dietary interventions to manage T2DM. Indeed, various strategies aimed at modifying the gut microbiome, including diets, prebiotics, probiotics, and pharmaceuticals, have been explored for T2DM management in recent years (5, 12, 13). Among these, metformin, a frontline treatment for T2DM, has been shown to modulate the gut microbiota, significantly increasing the abundance of *Akkermansia muciniphila* and reducing the presence of *Intestinibacter*, both of which are associated with improved metabolic outcomes (8).

In addition to metformin, traditional Chinese medicine (TCM), a form of polypharmacy, has been employed for years in China for the treatment of metabolic diseases (13). Recent extensive metagenomic studies have indicated that traditional Chinese herbs can treat T2DM by altering the gut microbiota (10, 14, 15). For instance, research by Xie Zhiyong on the herbal formula “Huanglian Jiedu Decoction” shows significant changes in gut flora, such as increasing the levels of beneficial *Akkermansia* bacteria while decreasing harmful *Firmicutes*, thus reducing insulin resistance (14). Similarly, Tong Xiaolin’s study on “Tianqi Capsule” reveals its ability to regulate blood sugar levels by enhancing the diversity of beneficial gut bacteria like *Bacteroidetes* and reducing *Proteobacteria*, known for their inflammatory properties (15).

In this study, we utilized a novel TCM formula, CCM, which has been patented (patent application publication no. CN 106214787 A). This formula consists of three herbal components: *Coptis rhizoma*, *Cinnamomi cortex*, and *Mume fructus*. It is based on the Jueyin theory, treating diabetes through a combined approach that targets both the liver and spleen (16). This herbal combination carefully considers the meridian tropism and properties of each herb, and their proportions are selected to ensure that the herbs work synergistically (17–20). This coordination helps regulate the liver and spleen, clears heat, and warms coldness, thereby exerting a therapeutic effect on diabetes (21).

Although our clinical practice has already demonstrated the significant efficacy of the herbal formula CCM in treating T2DM (1, 14, 15), the molecular mechanisms underlying this effect, particularly the role of the gut microbiota, remain unclear. To explore the correlation between gut microbiota and improvements in T2DM clinical indicators during CCM treatment, diabetic C57/db/db mice were administered the herb formula CCM at various concentrations for 4 weeks, using normal C57 mice as healthy controls and metformin as a drug-positive control for comparison.

Our investigation reveals that CCM outperforms metformin in T2DM management (especially in the middle and high dose groups) by significantly reducing hyperglycemia and hyperlipidemia, as well as showing superior results in oral glucose tolerance tests (OGTTs) and overall glucose management. Furthermore, CCM induces profound alterations in gut microbiota composition, notably enriching beneficial microbes linked to T2DM improvements, such as *Bacteroidetes* spp., *Akkermansia* spp., and *Parabacteroides* spp. These microbial changes strongly correlate with the observed clinical improvements in diabetic parameters, suggesting that the therapeutic effects of CCM are predominantly mediated through these microbiota modifications.

Furthermore, the gut microbial profiles and related functional profiles in different mouse groups were further tested for statistically significant associations with T2DM clinical parameters using MaAsLin2 (22), revealing that, compared to metformin, which primarily regulates blood lipids through gut microbiota, CCM also directly modulates blood glucose levels. Notably, all four microbial metabolic pathways significantly associated with lowering blood glucose levels were enriched only in the CCM-treated group. Among the 10 microbial metabolic pathways significantly associated with improvements in blood lipid parameters, five were enriched in both the CCM and metformin-treated groups, while the other five were unique to the CCM-treated group. These uniquely upregulated pathways in the CCM samples may, to some extent, explain their enhanced therapeutic effects compared to metformin. This also indicates that metformin and CCM improve and alleviate T2DM through different mechanisms of regulating gut microbiota structure and function.

In summary, this study reveals that CCM outperforms metformin in improving blood glucose, blood lipids, and body weight in T2DM model mice, especially in the middle- and high-dose groups. Furthermore, CCM induces distinct changes in gut microbiota and function, significant enrichment of three genera (*Bacteroides*, *Akkermansia*, and *Parabacteroides*) by over 40%, and specific functions such as chondroitin sulfate degradation, geraniol degradation, biotin biosynthesis, colanic acid building blocks biosynthesis, and the biosynthesis of vancomycin group antibiotics. Consequently, our findings identify potential probiotic candidates and functional pathways for T2DM treatment, further providing new therapeutic strategies.

## RESULTS

### High- and medium-dose CCM show greater treatment effect on hyperglycemia and hyperlipidemia than metformin in T2DM mice

**Fig 1:**
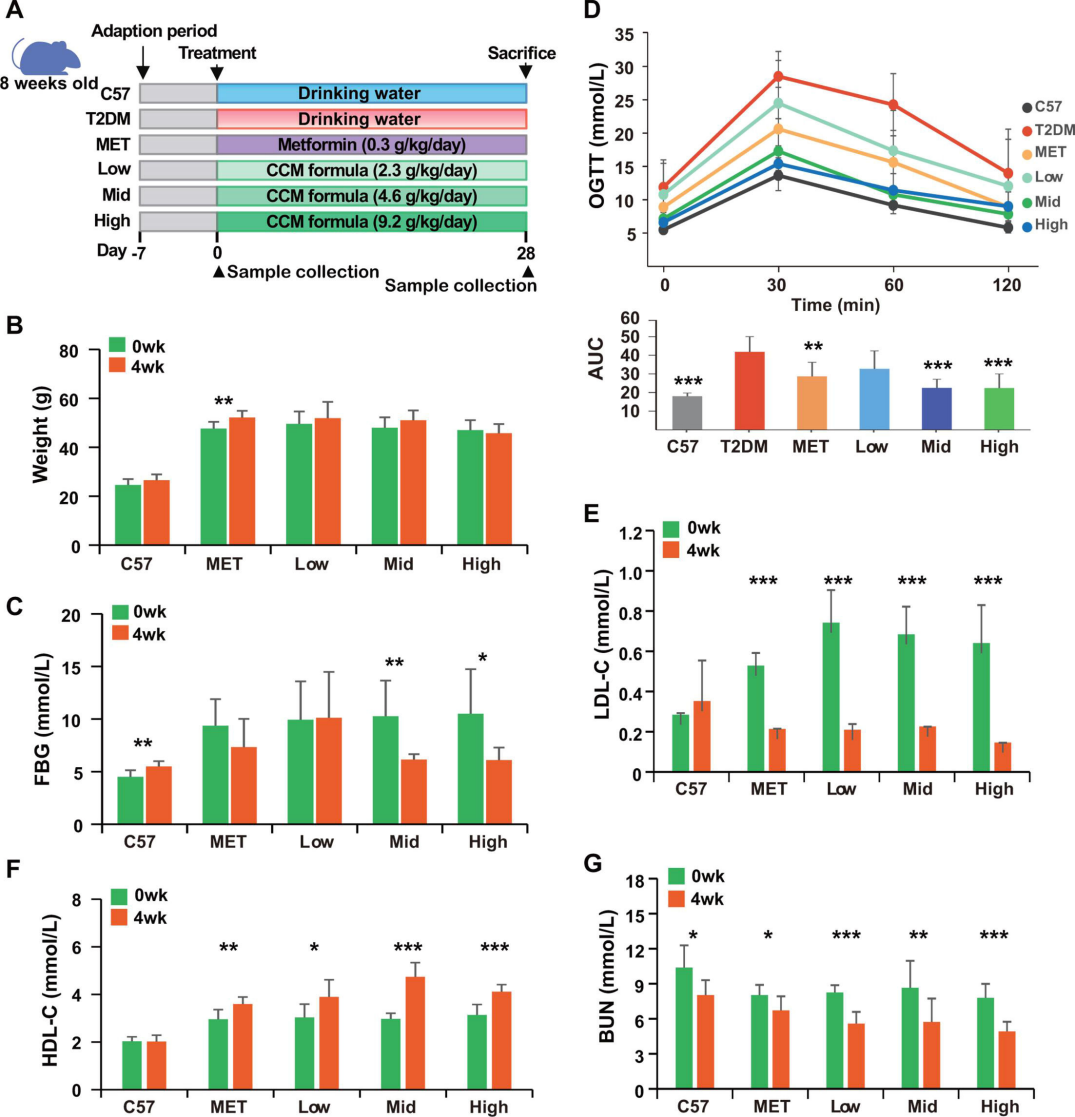
The effects of CCM and metformin treatment on body weight, FBG, OGTT, and blood lipid panels, including low-density lipoprotein cholesterol (LDL-C), high-density lipoprotein cholesterol (HDL-C), and blood urea nitrogen (BUN). (A) Schematic of the animal experiment; (B) body weight; (C) FBG; (D) blood glucose levels during the OGTT and AUC in OGTT; (E) LDLC; (F) HDLC; (G) BUN; Data are presented as means ± SD; Statistical significance: *P < 0.05, **P < 0.01, ***P < 0.001 (Wilcoxon test).

Body weight and glucose metabolism: The experimental design was shown in Fig. 1A. Over a 4 week period, we monitored the effects of CCM and metformin treatments on body weight and blood glucose metabolism in mice. The results showed that CCM treatments, particularly at medium and high doses, outperformed metformin in efficacy. Specifically, mice treated with CCM (Low, Mid, High dose groups) maintained their weight, whereas those in the metformin group (MET) exhibited a significant increase in weight (*P* = 0.0032) (Fig. 1B), suggesting a superior weight management effect with CCM. In terms of blood glucose control, both the Mid and High CCM groups showed a significant reduction in fasting blood glucose (FBG) levels (Mid group, *P* = 0.0024; High group, *P* = 0.022), while the metformin group displayed no substantial changes (Fig. 1C). Additionally, compared to the metformin group, the Mid and High CCM groups showed enhanced glucose clearance in the OGTT, as evidenced by lower peak glucose levels and reduced area under the curve (AUC) values (Fig. 1D).

Serum lipid profiles: Serum lipid profiles were also assessed over the same 4 week treatment period. Post-treatment, both the metformin and CCM groups significantly reduced serum low-density lipoprotein cholesterol (LDL-C) levels (*P* < 0.0001) (Fig. 1E). In contrast, high-density lipoprotein cholesterol (HDL-C) levels significantly increased in all treatment groups (MET *P* = 0.003, Low *P* = 0.019, Mid and High groups *P* < 0.0001) (Fig. 1F). Triglyceride (TG) levels remained unchanged across all groups (Fig. S1), with a noticeable increase in total cholesterol (T-CHOL) observed in the Mid and High groups, likely due to increased serum HDL-C levels (Fig. S1). While all treatment groups displayed some degree of curative effect, the Mid and High CCM groups showed greater efficacy in modifying lipid profiles compared to the metformin group.

In addition, we observed consistent results from the Hematoxylin-eosin(HE) stained liver sections (Fig. S2), which showed that the CCM compound effectively improved fat accumulation and fat degeneration in the livers of diabetic mice. The liver of normal mice showed orderly hepatocyte arrangement with no noticeable fat accumulation (Fig. S2A). The liver of diabetic mice typically showed fat degeneration, with clear fat droplets in the hepatocytes, resulting in vacuolated hepatocyte morphology (Fig. S2B). After metformin treatment, the overall hepatocyte morphology appeared more normal, with no significant fat degeneration, indicating that metformin helped regulate fat metabolism and improved liver condition (Fig. S2C). After treatment with a low dose of CCM, while some fat droplets were still present, there was an overall reduction in fat degeneration (Fig. S2D). The liver of diabetic mice treated with a high dose of CCM showed a more significant reduction in the number of fat droplets, and the hepatocyte morphology was closer to normal, showing the most significant improvement in reducing fat degeneration.

Liver and kidney parameters: Changes in serum alanine aminotransferase (ALT), aspartate aminotransferase (AST), blood urea nitrogen (BUN), and creatinine (CREA) levels were monitored as indicators of potential hepatic and renal injuries (2). No significant changes were observed in ALT, AST, and CREA levels. However, BUN levels consistently decreased post-treatment (*P* < 0.05) (Fig. 1G and Fig. S1), indicating that neither metformin nor CCM adversely affects liver and kidney functions.

### CCM herbal formula exerts a stronger impact on gut microbiota structure and their interactions than metformin in T2DM mice

To further investigate the structural changes in the gut microbiota of T2DM mice treated with metformin and the CCM herbal formula, we analyzed fecal samples using 16S rRNA gene sequencing. Normal C57 mice served as healthy controls. A total of 16,555,731 raw sequences (16,202 unique sequences) from 87 samples were obtained, with an average of 190,296 reads per sample.

**Fig 2:**
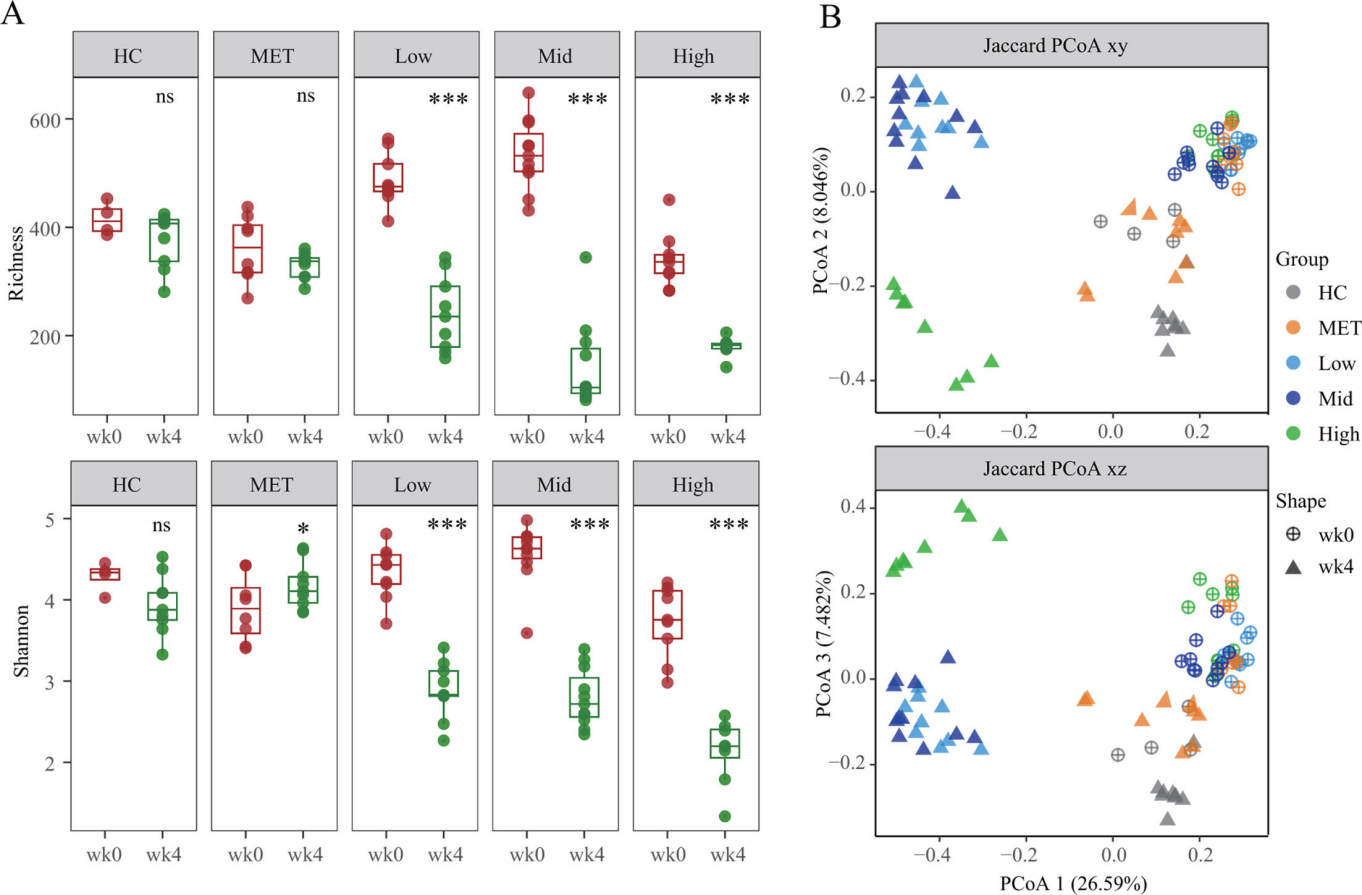
Dose-dependent alterations of the gut microbiota in T2DM mice treated with different doses of the CCM formula and metformin at weeks 0 and 4, using normal mice as healthy control. (A) The α-diversity of the Richness and Shannon values based on the operational taxonomic unit data from high-throughput sequencing. Statistical significance: *P < 0.05, **P < 0.01, ***P < 0.001, ns, significant (Wilcoxon test); (B) the β-diversity of gut microbiota based on PCoA using Jaccard distance matrix.

The α-diversity analysis of Richness and Shannon indices revealed a marked decrease in microbial diversity within the CCM-treated group (*P* < 0.001), in contrast to the increased diversity observed in the metformin-treated mice (Fig. 2A). Furthermore, β-diversity assessments using principal coordinate analysis (PCoA) and Jaccard distance highlighted significant shifts in gut microbiota structure post-treatment, with CCM showing a more substantial effect than metformin (Fig. 2B). Additionally, CCM intervention samples were distinctly clustered into two groups based on dosage variations, including the low- and middle-dose group (Low-wk4, Mid-wk4) and the high-dose group (High-wk4).

**Fig 3:**
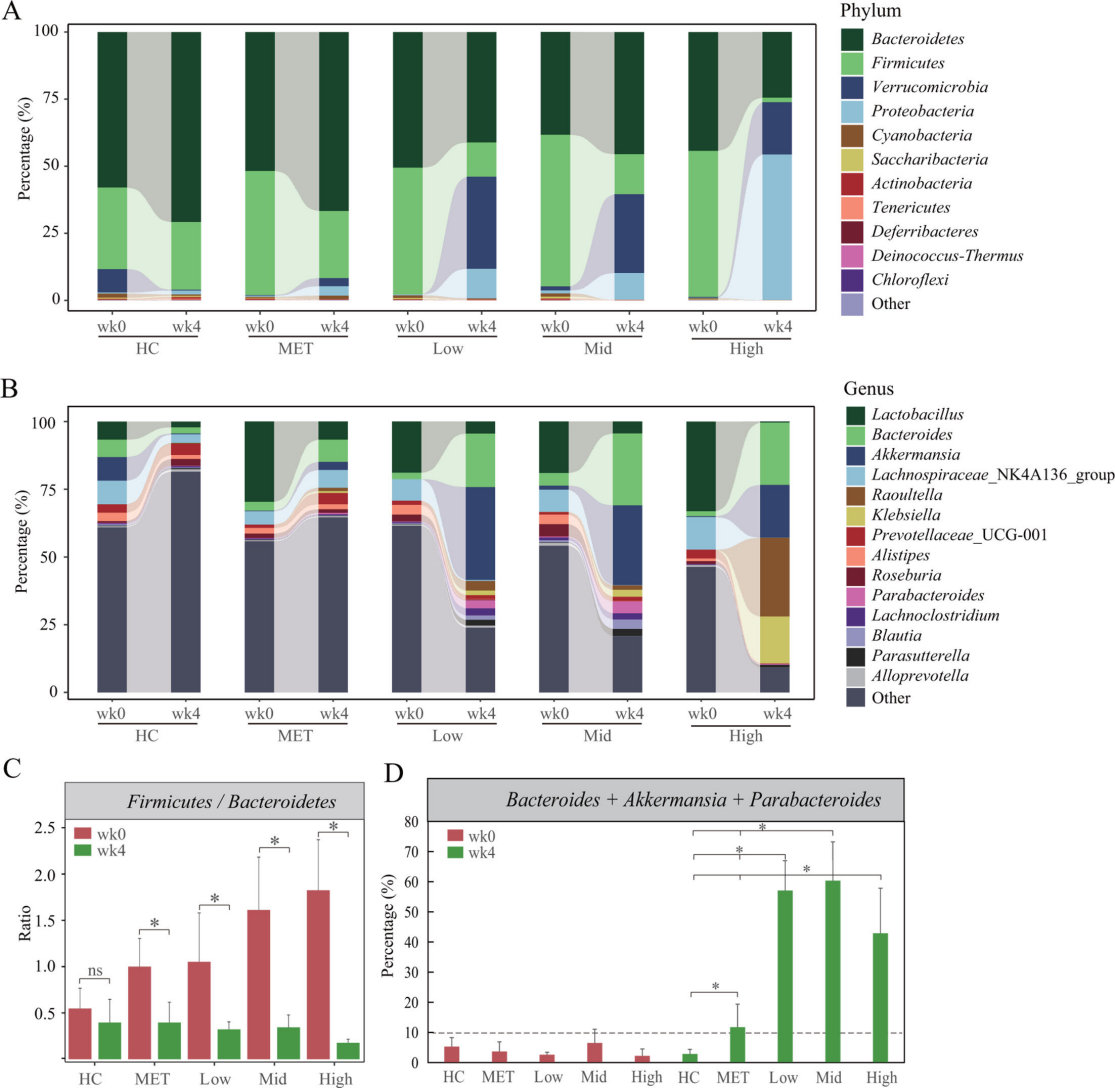
Taxonomic composition and alteration of the gut microbiota in T2DM mice treated with different doses of the CCM formula and metformin at weeks 0 and 4, using normal mice as healthy control. (A) Relative abundances of gut microbiota at the phylum level; (B) relative abundances of gut microbiota at the genus level; (C) the ratio of *Firmicutes* to *Bacteroidetes*; (D) the main genera stimulated by CCM and their abundance in different groups. Statistical significance: *P < 0.05, ns, significant (Wilcoxon rank sum test).

Our detailed analysis of the changes in gut microbiota composition before and after treatment at both the phylum and genus levels confirmed the stronger influence of the CCM herbal formula on the gut microbiota of T2DM mice. At the phylum level (Fig. 3A and Fig. S3), the gut microbiota predominantly consisted of *Firmicutes* and *Bacteroidetes*. Post-treatment, there was a significant decrease in the *Firmicutes/Bacteroidetes* ratio (*P* < 0.01) (Fig. 3C), a marker often linked to gut microbial imbalance associated with obesity (23, 24). Previous studies also indicated a higher *Firmicutes*/*Bacteroidetes* ratio in T2DM mice (2, 11). This trend was observed in both the metformin and CCM groups, but the reduction was more pronounced in the CCM groups, particularly the Mid and High dosage groups, indicating a stronger impact of CCM. Moreover, the abundance of the phylum *Verrucomicrobia* exhibited a significantly greater increase in the CCM-treated groups compared to the metformin-treated group.

At the genus level, we identified 124 genera, with 14 dominating (abundances above 2%) (Fig. 3B and D and Fig. S3). Notably, Fig. 3 underscores the greater influence of the CCM herbal formula on the gut microbiota composition of T2DM mice compared to metformin. This is particularly evident in the remarkable augmentation of genera such as *Akkermansia*, *Bacteroides*, and *Parabacteroides* in the CCM-treated groups (Fig. 3D).

**Fig 4:**
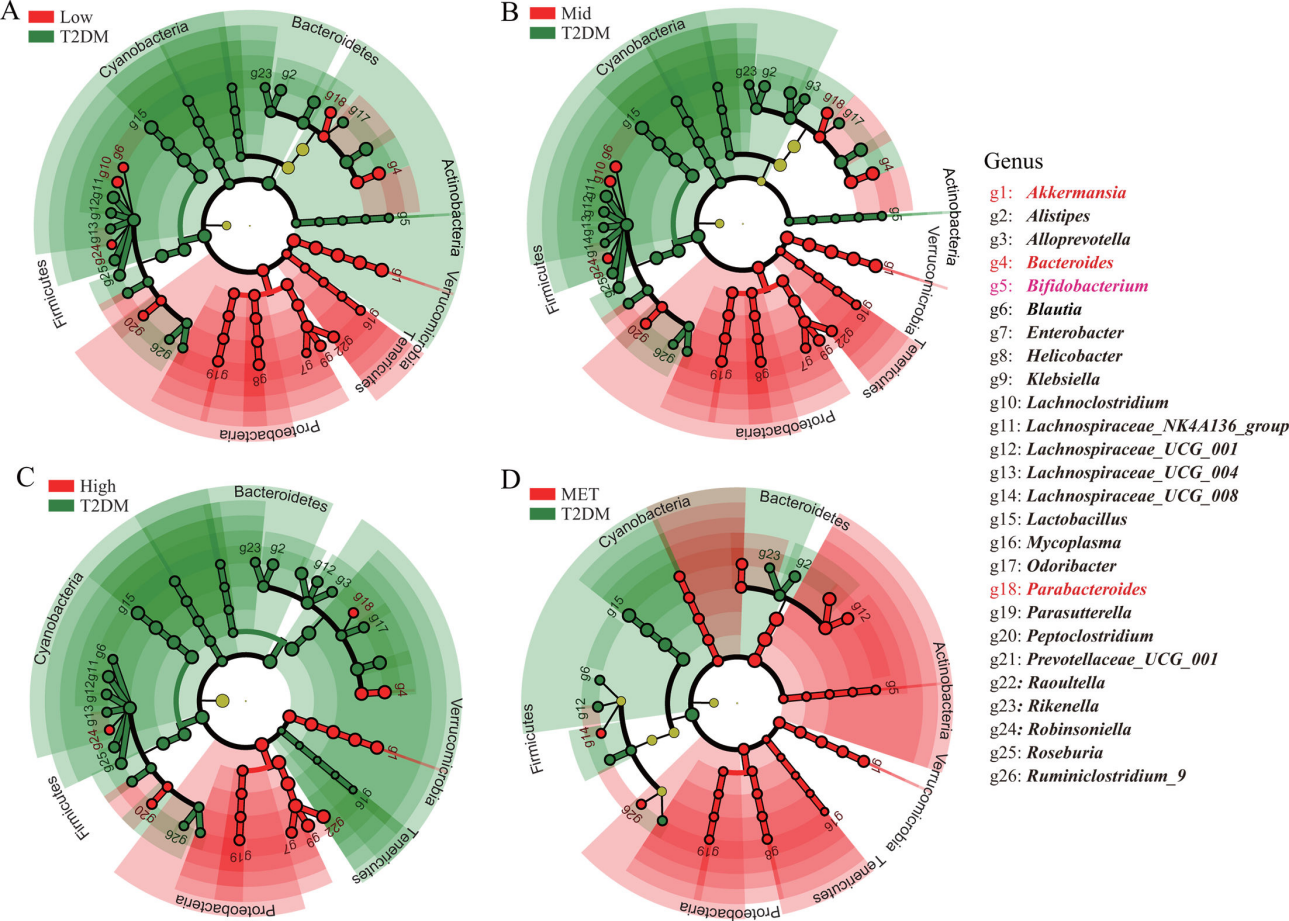
LEfSe identification of the significantly differential bacterial taxa in T2DM mice at week 0 (red) versus T2DM mice after drug treatment at week 4 (green). (A) The CCM Low group versus T2DM group; (B) the CCM Mid group versus T2DM group; (C) the CCM High group versus T2DM group; (D) the MET group versus T2DM group. Significantly changed genera are listed in the legend on the right (*P* < 0.05). Genera enriched in both the CCM groups (Low, Mid, High) and the metformin (MET) group are marked in red, while the genera specially enriched in the MET group are marked in pink.

Using Linear discriminant analysis Effect Size analysis (LEfSe) (25), we identified distinct genus differences between the treatment groups. For the analysis, diabetic C57/db/db mice samples at week 0 (MET/Low/Mid/High wk0) were combined into the T2DM group based on β-diversity (Fig. 2B). As shown in Fig. 4, the CCM-treated groups, regardless of dosage, were significantly enriched in genera such as *Bacteroides*, *Parabacteroides*, *Blautia*, *Raoutaella*, *Klebsiella*, *Parasutterella*, *Robinsoniella*, and *Akkermansia* (Fig. 4A through C). The metformin group exhibited enrichment in some of the same genera as the CCM groups, including *Bacteroides*, *Parabacteroides*, *Parasutterella*, and *Akkermansia* (Fig. 4D). Notably, *Bifidobacterium* was particularly abundant in the metformin group, aligning with reports that metformin promotes the growth of *Bifidobacterium adolescentis in vivo* and *in vitro* (8). Additionally, a multi-group comparison using the Wilcoxon test (Fig. S4) revealed a significantly higher abundance of *Bifidobacterium*, *Ruminiclostridium*, *Bacteroidales*, and *Allobaculum* in the healthy control group, while *Odoribacter*, *Rikenella*, *Lactobacillus*, *Peptococaceae*, and *Roseburia* were more prevalent in the T2DM group.

**Fig 5:**
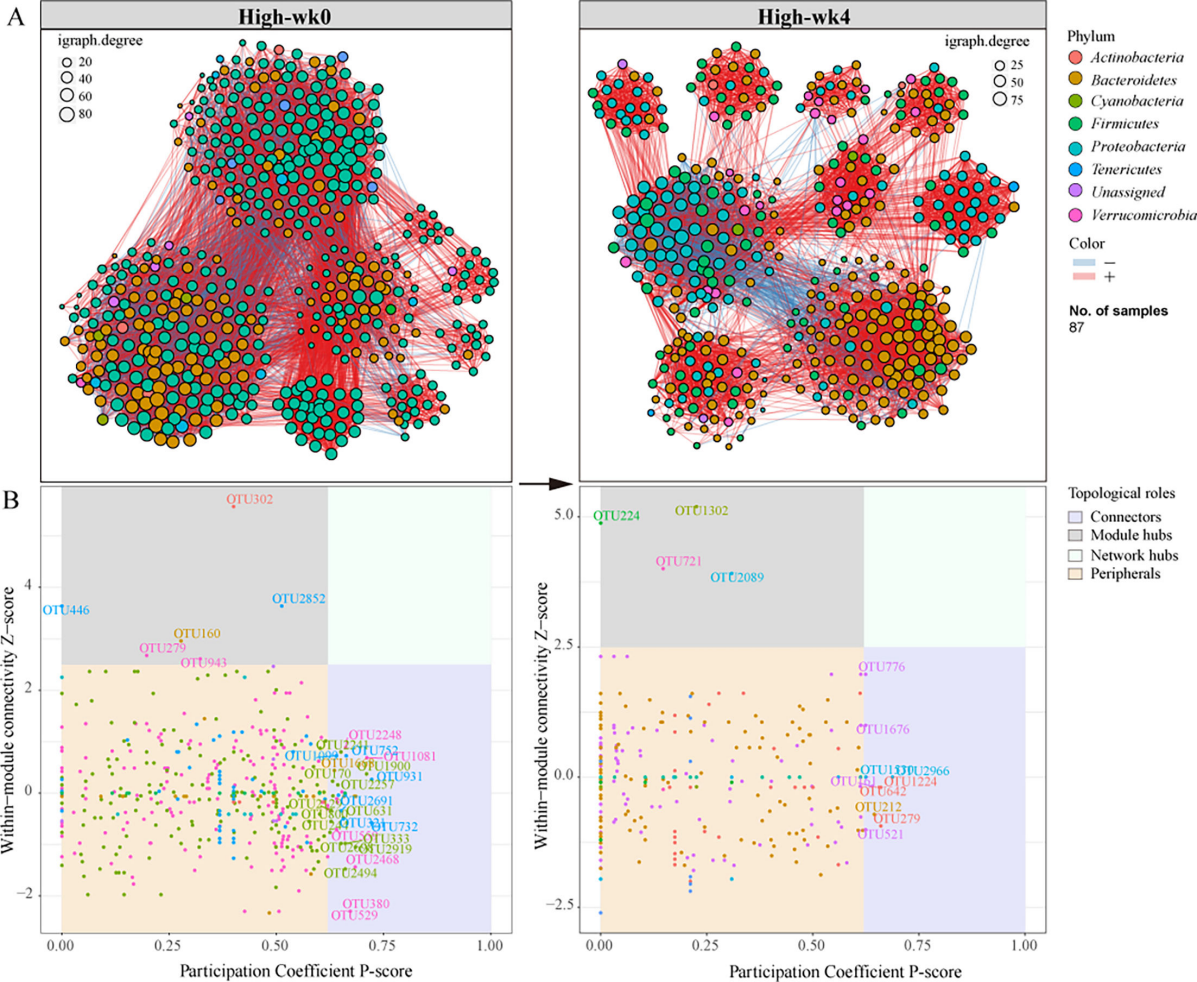
Co-abundance networks of the gut microbiota in T2DM mice treated with a high dose of CCM at weeks 0 and 4. (A) Co-abundance networks, constructed using all OTUs in each group. Negatively related OTUs are linked by blue lines, and positively related OTUs are linked by red lines; (B) the topological roles of OTU nodes in the networks, which are grouped into four categories based on their within-module connectivity (Z-score) and participation coefficient (P-score) among modules.

To understand the global changes in microbial interactions due to drug treatment, we constructed co-abundance networks using all operational taxonomic units (OTUs) from each group. As depicted in Fig. 5A, Fig. S5, and Table 1, we observed an increase in modularity within the gut bacterial networks post-treatment, particularly pronounced in the CCM-treated groups. These groups exhibited enhanced network parameters, including average path length, diameter, and centralization metrics like betweenness and closeness.

**TABLE 1:** Topological properties for the co-abundance networks for the gut microbiome in different groups

Network parameters	MET		Low		Mid		High	
	MET-wk0	MET-wk4	Low-wk0	Low-wk4	Mid-wk0	Mid-wk4	High-wk0	High-wk4
Average path length	1.704	1.719	1.641	1.692	1.423	1.543	1.850	2.226
Diameter	3.780	4.282	3.485	3.620	3.114	3.982	3.606	4.702
Clustering coefficient	0.609	0.681	0.457	0.676	0.485	0.848	0.507	0.670
Centralization degree	0.213	0.212	0.197	0.222	0.172	0.284	0.094	0.144
Centralization betweenness	0.013	0.034	0.008	0.060	0.006	0.033	0.029	0.120
Centralization closeness	0.229	0.217	0.193	1.123	0.181	1.042	0.130	0.227

We further classified the topological roles of OTU nodes within these networks into four categories based on their within-module connectivity (Z-score) and participation coefficient (P-score) among modules. These categories included peripherals, connectors, module hubs, and network hubs (26, 27). The Zi-Pi plot (Fig. 5B and Fig. S5) demonstrated that module hubs and connectors were predominantly associated with families such as *Ruminococcaceae*, *Lachnospiraceae*, and the *Bacteroidales*_S24-7 group. Interestingly, no network hubs were identified. A key observation was the notable reduction in the number of connectors following treatment, particularly after CCM intervention. This reduction, potentially linked to the significant decrease in families like *Ruminococcaceae* and *Lachnospiraceae* (Fig. S5), indicates that CCM treatment more profoundly alters the interconnectedness and structure of the gut microbiota than metformin, potentially affecting the overall microbial function and dynamics in T2DM mice.

### CCM and metformin exert differential effects on T2DM clinical indicators by activating distinct OTUs and their associated microbial modules

**Fig 6:**
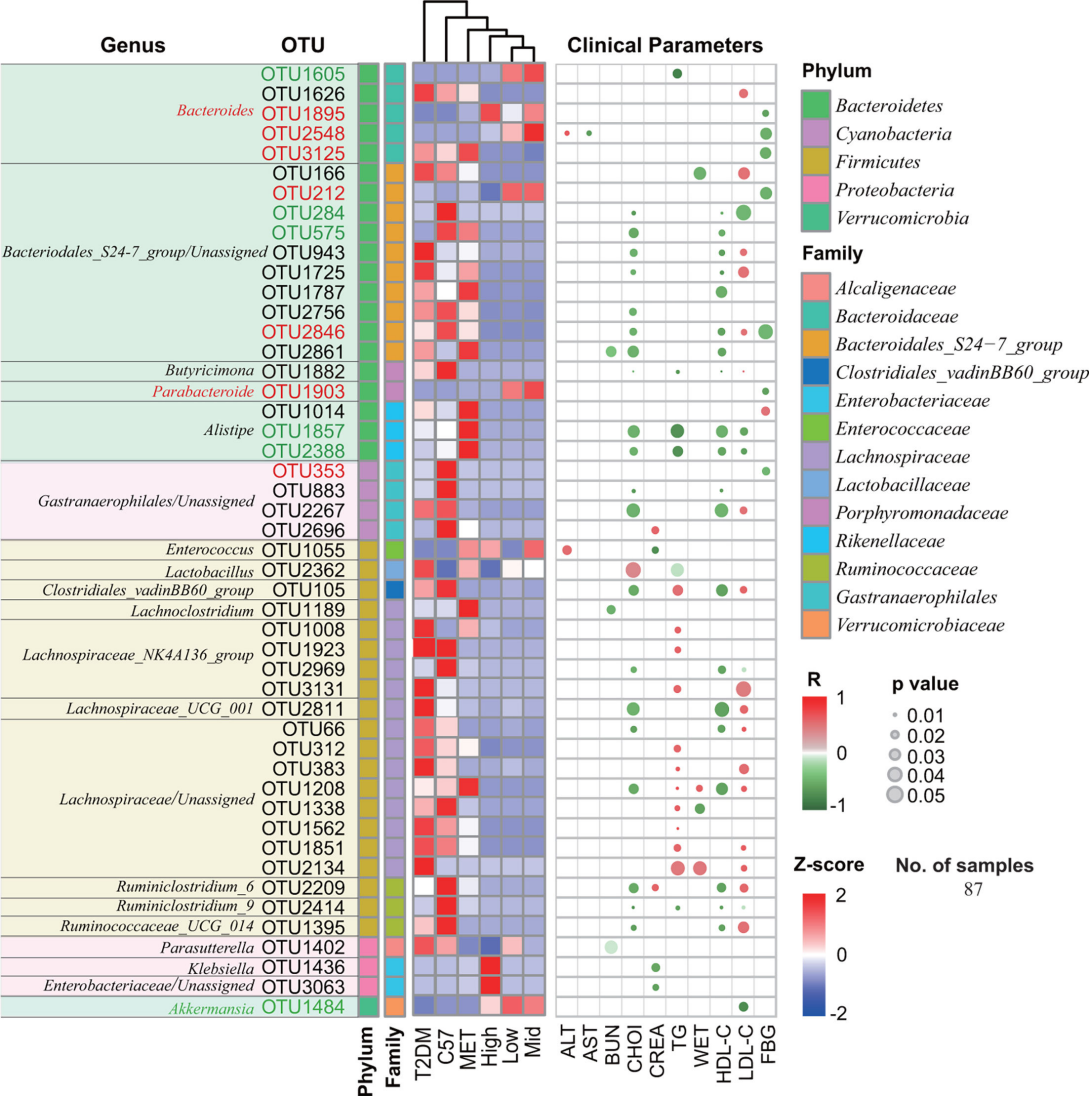
Association of biomarkers (differential OTUs) with T2DM clinical parameters. Relative abundances of the 48 differential OTUs in different groups are presented in a heatmap based on Z-score. The associations were evaluated using MaAsLin2 with a linear multivariate regression model. The *R*-value represents the ratio of covariance to standard deviations between two variables, and associations with *P* < 0.05 are presented. Representative OTUs correlated with the decrease of FBG are marked in red, and OTUs correlated with the decrease of LDL-C are marked in green.

To identify the specific species correlating with metformin and CCM treatments in T2DM mice, we conducted a differential OTU analysis between the T2DM group and the treatment groups (MET/Low/Mid/High-wk4) using edgeR, STAMP, and LEfSe (25, 28, 29). Merging results from these methods, we identified 613 differential OTUs, accounting for over 95% of total abundance (Table S1). Subsequent analysis with MaAsLin2 (22) revealed 48 key OTUs significantly associated with at least one T2DM clinical parameter (Fig. 6).

Among these OTUs, seven showed a negative correlation with FBG levels, including strains from *Bacteroides* (*n* = 3), *Bacteroidales_S24-7* group (*n* = 2), *Parabacteroide* (*n* = 1), and *Gastranaerophilales* (*n* = 1). Six OTUs correlated with a decrease in LDL-C, including *Bacteroidales_S24-7* group (*n* = 1), *Alistipes* (*n* = 2), *Lachospiraceae*_NK4A136 (*n* = 1), *Ruminiclostridium*_9 (*n* = 1), and *Akkermansia* (*n* = 1). Two OTUs were linked to weight reduction, including *Bacteroidales_S24-7* group (*n* = 1) and *Lachospiraceae* (*n* = 1). Additionally, three OTUs negatively correlated with BUN levels, including *Parasutterella* (*n* = 1), *Lachnoclostridium* (*n* = 1), and *Bacteroidales_S24-7* group (*n* = 1).

In our assessment of drug treatment effects on OTUs and clinical features, distinct patterns emerged. In the metformin-treated group, the activated OTUs were primarily linked to alterations in blood lipid profiles. For example, OTUs from the *Bacteroidales S24-7* group (OTU 575, OTU 725, and OTU 1787) and the *Alistipes* genus (OTU 1857 and OTU 2388) demonstrated significant correlations with changes in HDL-C and LDL-C levels. In contrast, in the CCM-treated groups, the activated microbial communities showed significant associations with the improvements in both blood glycemic and lipid parameters in diabetic mice. This includes specific OTUs from th*e Bacteroides* genus (OTU 1895, OTU 2548, and OTU 3125), and OTU 1903 from *Parabacteroides*, which were significantly linked to the reduction of FBG levels. The OTU 1484 from the *Akkermansia* genus, which was remarkably stimulated in the CCM-treated groups, showed a notable association with the decrease in LDL-C levels.

**Fig 7:**
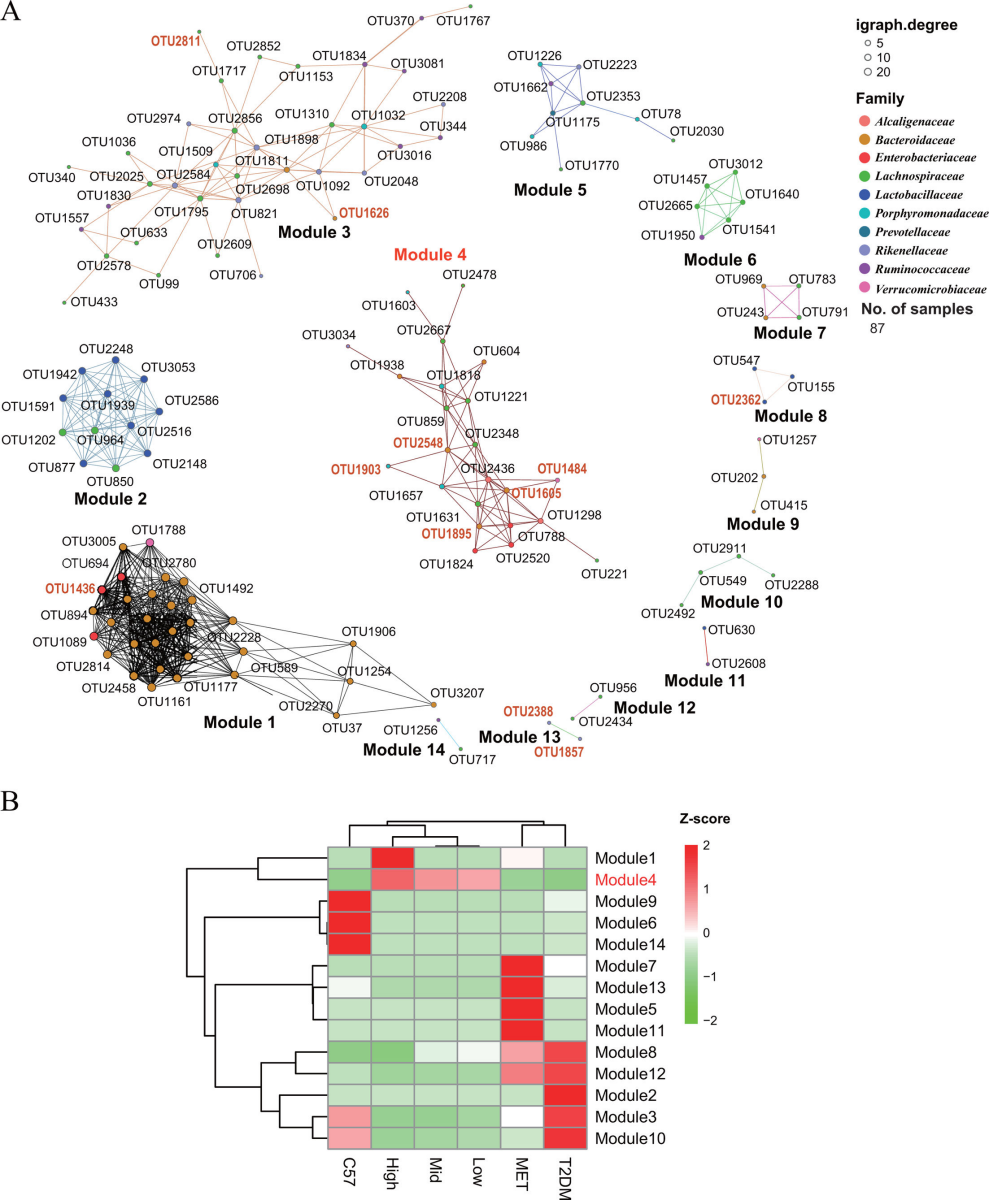
Key co-abundant microbial modules (groups) and their relative abundance in different groups. (A) The co-abundant modules, reconstructed based on the obtained differential OTUs. The *R*-values (Spearman correlation) >0.8 and *P*-values <0.05 are shown in the figure. OTUs significantly associated with T2DM clinical parameters are marked in orange. (B) The relative abundance of modules in different groups.

Network modules can reflect closely associated species involved in metabolic processes such as syntrophy (26). To gain insights into the interacting functional microbes within the gut microbiome, we constructed a co-abundance network with high modularity using the 613 OTUs. These OTUs clustered into 14 modules based on their strong Spearman correlations (*R* > 0.8) (Fig. 7). The upregulated and downregulated modules between MET and CCM treatment groups were distinctly different, indicating their varied impacts and mechanisms of action on the gut microbiota structure in T2DM mice. Post-CCM treatment, two modules (modules 1 and 4) significantly increased. Module 1, predominantly consisting of species from *Bacteroidia* (*n* = 17) and *Gammaproteobacteria* (*n* = 3), was notably abundant in the high-dose CCM group. Module 4, the most diverse module with 12 different genera (such as *Alistipes*, *Bacteroides*, *Parabacteroides,* and *Blautia*), significantly increased across all CCM-treated groups (Fig. S6A). Within module 4, five OTUs were significantly linked to T2DM clinical parameters (Fig. S6B): three negatively associated with FBG levels (OTU 2548, 1903, and 1895), one with LDL-C (OTU 1484), and one with TG levels (OTU 1605). This suggests that module 4 may be a key functional module in the CCM intervention groups. Post-MET treatment, four modules (modules 7, 13, 5, and 11) significantly increased. However, within these modules, only two OTUs, from *Bacteroides* and *Lachnospiracea*_UCG_001 respectively (OTU1626 and OTU2811), were significantly correlated with the clinical indicators (LDL-C and HDL-C).

Although the metformin-induced microbial modules (modules 7, 13, 5, and 11) differed from those in the CCM groups (modules 1 and 4), the suppressed modules were similar, predominantly involving five significantly increased modules in the T2DM group (8, 12, 2, 3, and 10). The suppressed modules, primarily consisting of *Bacilli* and *Clostridia*, were common to both treatments but showed a larger decline in the CCM group. This significant decrease in the CCM group suggests a more substantial reshaping and rebuilding of the gut microbiota structure in T2DM mice compared to the MET group, potentially offering distinct therapeutic mechanisms.

### Enhanced activation of metabolic pathways in CCM-treated T2DM mice compared to the MET group

**Fig 8:**
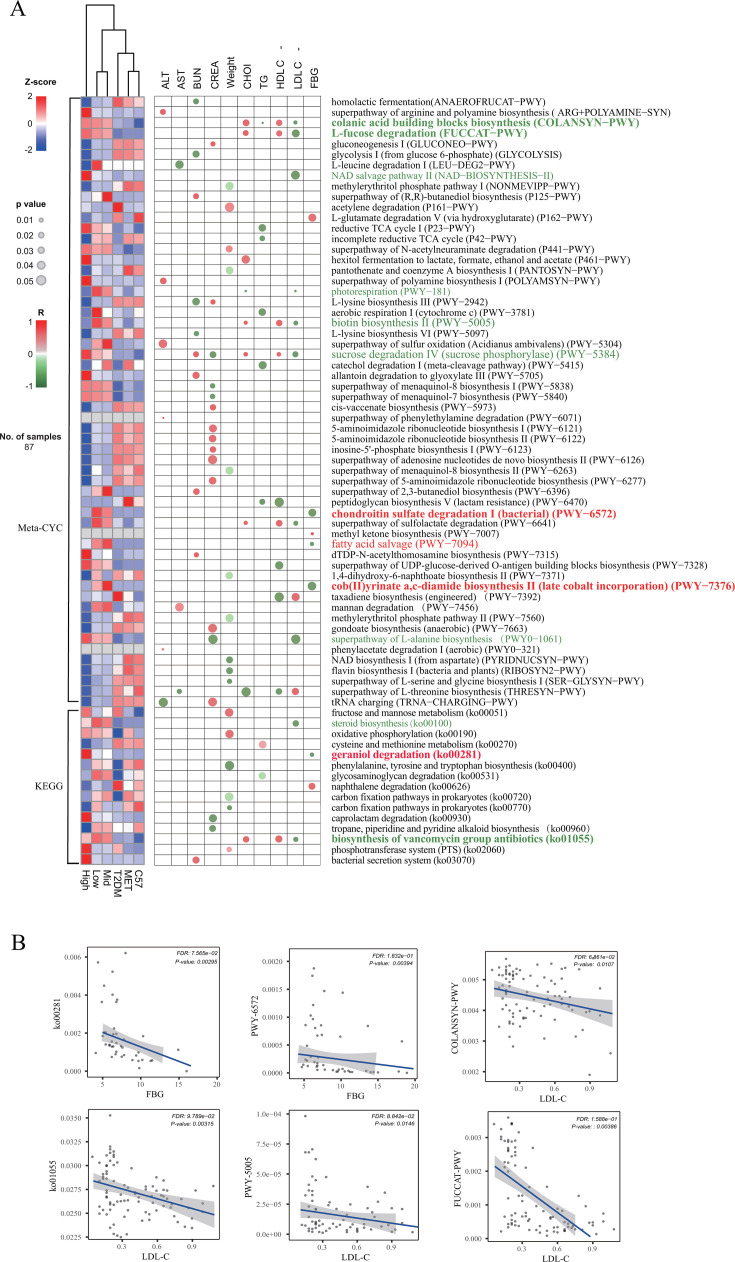
Association of microbial metabolic pathways with T2DM clinical features. (A) Relative abundances of the microbial metabolic pathways in different groups are presented in a heatmap. The associations were evaluated using MaAsLin2 with a linear multivariate regression model. The *R*-value represents the ratio of covariance to deviations between two variables, and associations with *P* < 0.05 are presented. Pathways correlated with the decrease of FBG are marked in red, and those correlated with the decrease of LDL-C are marked in green. (B) Selected significant associations of clinical features with microbial metabolic pathway abundances. Nominal *P*-values and False Discovery Rate (FDR)-corrected q-values are assigned by MaAsLin2.

Utilizing PICRUSt2, which employs a machine learning algorithm to predict the functional profiles of microbial communities from 16S rRNA gene sequences (30), we predicted gut microbiota functions in our study (Fig. 8). A total of 147 and 365 microbial metabolic pathways were identified based on the KEGG and MetaCYC databases, respectively. These pathways were then analyzed for significant associations with T2DM clinical parameters using MaAsLin2, identifying 71 key pathways correlated with at least one clinical parameter (*P* < 0.05) (Fig. 8 and Fig. S7).

Notably, four bacterial metabolic pathways were negatively correlated with FBG: geraniol degradation (ko00281), fatty acid salvage (PWY-7094), chondroitin sulfate degradation I (PWY-6572), and Cob(II)yrinate a, c-diamide biosynthesis II (PWY-7376). These findings align with previous research highlighting the potential role of chondroitin sulfate in the development of insulin resistance, a hallmark of T2DM (31, 32). Additionally, Cob(II)yrinate a, c-diamide, a vitamin B12 precursor (33), has been linked to T2DM risk (34). Beyond FBG, 10 microbial pathways showed significant associations with improvements in blood lipid parameters, such as reducing LDL-C and TG while increasing HDL-C. These pathways include Steroid biosynthesis (ko00100), Biosynthesis of vancomycin group antibiotics (ko01055), Sucrose degradation IV (PWY-5384), L-fucose degradation I (FUCCAT-PWY), Biotin biosynthesis II (PWY-5005), Colanic acid building blocks biosynthesis (COLANSYN-PWY) Photorespiration I (PWY-181), Sulfolactate degradation (PWY-6641), NAD salvage pathway II (to nicotinamide riboside), and Homogalacturonan biosynthesis (PWY0-1061). Prior studies have reported the benefits of biotin supplementation on FBG, LDL-C, and TG in T2DM patients (35). Furthermore, dietary L-fucose, a prebiotic metabolized by gut bacteria, has been shown to positively affect cholesterol and TG levels (36, 37).

It is noteworthy that these metabolic pathways, significantly associated with glucose and lipid improvement, are predominantly activated in the CCM-treated groups rather than in the MET group. Figure 8 illustrates that of the four pathways integral to the FBG indicator, PWY-7094 and PWY-7376 register the highest abundance in the CCM Mid group. Additionally, ko00281 is most abundant in the CCM High group, and PWY-6572 is predominant in the CCM Low group. Similarly, pathways associated with lipid indicator improvement are more actively favored in CCM-treated groups over the MET group. Notably, PWY0-1061, PWY-5384, and FUCCAT-PWY show the highest abundance in the CCM High group. These observations explain the superior therapeutic efficacy of CCM in T2DM mice compared to metformin to some extent, particularly in the High and Mid treatment groups.

## DISCUSSION

In TCM, T2DM falls under the category of “Xiaoke” (relieving thirst) and is primarily attributed to Qi and Yin deficiency, leading to imbalances in the spleen, gut, and liver. These imbalances contribute to internal heat (inflammation), blood stasis, phlegm dampness, and liver cold (insulin resistance) (5). The TCM theory highlights the importance of spleen, gut, and liver in maintaining metabolic homeostasis (38–40). CCM, composed of *Coptis rhizoma, Cinnamomi cortex,* and *Mume fructus,* is designed to restore systemic balance based on TCM principles. *Coptis rhizoma (Huanglian),* derived from the rhizome of *Coptis chinensis, Coptis deltoidea,* or *Coptis teeta*, is traditionally used to clear intestinal heat and dry dampness, with established applications in treating diabetes, pertussis, aphthous ulcers, bacillary dysentery, and eczema in ancient China (18). *Cinnamomi cortex* (*Cinnamon bark*) warms the liver, relieves stagnation, and has been shown to reduce abdominal fat and enhance intestinal antioxidant capacity (16, 41). *Mume fructus* (the fruit of *Prunus mume*) promotes body fluid production, relieves thirst, and harmonizes the spleen and liver function (42).

In the study, we have demonstrated the notable efficacy of the traditional Chinese herbal formula CCM in treating T2DM in a mouse model, using metformin as a benchmark. Our findings indicate that CCM not only regulates blood glucose and lipid levels more effectively but also manages body weight better than metformin (Fig. 1). Particularly noteworthy is the significant alteration in gut microbiota composition induced by CCM treatment. Furthermore, we have shown that some distinctive enriched bacterial genera and functions in CCM-treated groups are correlated with the T2DM phenotypes, suggesting that CCM has a distinct impact on microbial communities compared to metformin (Fig. 2 and 3). These enriched genera and functions provide potential probiotics for treating T2DM and offer a novel therapeutic strategy.

First, our study underscores the significant roles of specific gut bacteria—*Bacteroides, Akkermansia* and *Parabacteroides*—in managing T2DM. These bacteria were significantly enriched and considerably more abundant in CCM-treated mice, accounting for over 50% of the gut microbiota (*Bacteroides*: 23.1%, *Akkermansia*: 27.7%, *Parabacteroides*: 2.6%) compared to lower percentages (*Bacteroides*: 3.73%, *Akkermansia*: 4.04%, *Parabacteroides*: 11.75%) in diabetic C57/db/db, healthy, and metformin-treated mice (Fig. 3D). Moreover, these bacteria were found to be negatively associated with blood glucose and lipid levels (Fig. 6). Further analysis of specific OTUs revealed that OTUs from *Bacteroides* (OTU 1895, OTU 2548, and OTU 3125) and *Akkermansia* (OTU 1484) showed strong negative correlations with FBG and LDL-C levels, supporting the beneficial roles of these bacteria in T2DM treatment. Specifically, *Bacteroides* OTU 1895 and OTU 2548 were linked to a reduction in FBG, while *Akkermansia* OTU 1484 correlated with decreased LDL-C levels. Additionally, *Parabacteroides* OTU 1903 was also associated with a reduction in FBG, further reinforcing the therapeutic potential of these OTUs in improving blood glucose control.

Previous research has documented the therapeutic effects of these bacteria on T2DM, particularly their capacity to lower blood glucose and lipid levels (16, 43, 44). A comprehensive review of 42 studies also confirmed the inverse relationship between *Bacteroides* and *Akkermansia* and T2DM (45). Additionally, *Parabacteroides distasonis* has also been demonstrated to enhance glucose-lipid metabolism and alleviate T2DM by producing beneficial metabolites such as succinate and secondary bile acids (43). Furthermore, *Akkermansia muciniphila*, a standout bacterium in T2DM treatment, plays a crucial role in improving intestinal barrier integrity and reducing lipogenesis and gluconeogenesis, further establishing its significance in T2DM management (8, 22, 44, 46). These findings emphasize the pronounced impact of CCM treatment in elevating the abundance of these beneficial bacteria compared to metformin, highlighting their crucial and special role in reshaping the gut microbiome to support T2DM management.

We subsequently explored the impact of CCM and metformin treatments on the ecological networks of the gut microbiota, noting distinct influences on network modules (Fig. 7). Post-treatment analyses showed significant activation of module 4 across all CCM-treated groups. This co-abundance network module includes various OTUs from beneficial genera, including the previously mentioned *Bacteroides*, *Akkermansia*, and *Parabacteroides*, which were among the top 6 enriched genera (21.3%, 12.1% and 1.7% respectively). Additionally, the other three genera—*Blautia*, *Raoutella*, and *Enterobacter*—also showed significantly co-enriched rankings fourth to sixth (1.2%, 8.8% and 0.6%, respectively). Notably, *Blautia* has been reported as a beneficial microbe for managing diabetes with hyperlipidemia (1). However, *Raoutella* and *Enterobacter*, typically considered opportunistic pathogens, have not been reported to have direct associations with T2DM. Despite this, they demonstrated significant co-enrichment with beneficial microbes such as *Bacteroides*, *Akkermansia*, and *Parabacteroides* (OTUs 2548, 1903, 1895, and 1484), suggesting potential ecological roles for these activated microorganisms by CCM: although not directly linked to clinical outcomes, they may contribute to creating a symbiotic environment that supports the growth and function of beneficial bacteria.

The unique chemical composition of CCM may contribute to the significant changes in gut microbiota observed in the CCM-treated groups. Berberine, the primary active component of *Coptidis rhizome* (1), has been shown to promote the growth of *Akkermansia muciniphila*, which is consistent with the findings of this study (47). Additionally, berberine has been reported to improve blood glucose levels by increasing short-chain fatty acid (SCFA)-producing bacteria such as *Faecalibacterium*, *Butyricimonas, Clostridium XIVa*, *Butyricicoccus*, *Parabacteroides*, and *Roseburia* (48). *Cinnamomi cortex* contains over 300 chemical compounds, including cinnamaldehyde, polyphenols, diterpenes, flavonoids, and polysaccharides. Among these, cinnamaldehyde exhibits anti-inflammatory and antioxidant properties that may influence gut microbial metabolism. A recent study demonstrated that cinnamon oil, rich in cinnamaldehyde, significantly altered the gut microbiota in rats by decreasing the abundance of conditional pathogens such as *Eubacterium fissicatena* group while increasing beneficial bacteria like *Roseburia* (41). *Mume fructus*, rich in organic acids and polyphenols, contributes to metabolic regulation via microbial metabolism. While most organic acids in *Mume fructus* are poorly absorbed, they are metabolized by gut bacteria into SCFAs, which regulate glucose and lipid metabolism. *Mume fructus* has also been shown to significantly alter gut microbiota composition and alleviate intestinal barrier damage and inflammation in rats. Enrichment of *Blautia* (also observed in the CCM group) and *Turicibacter* contributes to its therapeutic effects, including the treatment of colitis (49). These findings highlight the critical role of CCM’s chemical components in modulating gut microbiota to exert therapeutic effects in T2DM.

The study further investigated the relationship between changes in microbial metabolic functions and the alleviation of T2DM (Fig. 8). Compared to metformin, which primarily regulates blood lipids through gut microbiota, CCM not only regulates blood lipids but also directly modulates blood glucose levels. This reveals the different mechanisms through which CCM and metformin regulate T2DM via gut microbiota. Specifically, four distinct metabolic pathways associated with decreased blood glucose levels were identified exclusively in the CCM-treated groups (highlighted in red, Fig. 8). These pathways, enriched in the CCM-treated groups, were found to be negatively associated with FBG levels and involved chondroitin sulfate (CS) degradation (PWY-6572), geraniol degradation (ko00281), and vitamin B12 biosynthesis (PWY-7376). Elevated CS levels in the kidneys of T2DM patients and the CS component of the urinary trypsin inhibitor have been suggested as potential markers for T2DM (32). In diabetic rats, oral geraniol was found to improve various parameters related to glucose metabolism (50). Additionally, Fogelman et al. documented a significant correlation between vitamin B12 levels and insulin treatment in T2DM patients (34). Besides these pathways, the fatty acid salvage pathway (PW-7094), enriched in the CCM-treated groups, was found to be negatively associated with FBG, a finding not previously reported in other literature.

In addition to blood glucose, 10 microbial pathways were identified as significantly associated with improvements in blood lipid parameters (Fig. 8). Among these, five pathways were enriched in both CCM- and metformin-treated groups compared to the T2DM groups, including steroid biosynthesis (ko00100), biosynthesis of vancomycin group antibiotics (ko01055), NAD salvage pathway II (to nicotinamide riboside), L-fucose degradation I (FUCCAT-PWY), and the superpathway of L-alanine biosynthesis (PWY01061). The degradation of L-fucose (FUCCAT-PWY) by gut microbes correlated with reduced cholesterol, triglycerides, and LDL-C in rats (37). A novel link was observed between the biosynthesis of vancomycin group antibiotics (ko01055) and improvements in LDL-C and HDL-C levels. Vancomycin’s selective inhibition of most gram-positive bacteria may potentially affect the *Firmicutes*/*Bacteroidetes* ratio, thereby influencing lipid levels (51).

Besides the shared microbial pathways, five pathways were identified as enriched exclusively in the CCM-treated groups: sucrose degradation IV (PWY-5384), biotin biosynthesis II (PWY-5005), colanic acid building blocks biosynthesis (COLANSYN-PWY), photorespiration I (PWY-181), and sulfolactate degradation (PWY-6641). Prior studies have reported the benefits of biotin supplementation on FBG, LDL-C, and TG in T2DM patients (35). Although no direct evidence yet supports a causal relationship between colanic acid and T2DM alleviation, its biosynthesis was significantly correlated with lipid profile changes, suggesting its role as a probiotic produced by intestinal bacteria (52).

Overall, we found that all four microbial metabolic pathways significantly associated with lowering blood glucose levels were enriched only in the CCM-treated group. Among the 10 microbial metabolic pathways significantly associated with improvements in blood lipid parameters, five were enriched in both the CCM- and metformin-treated groups, while the other five were unique to the CCM-treated group. These uniquely upregulated pathways in the CCM samples may, to some extent, explain their enhanced therapeutic effects compared to metformin. Our results suggest that CCM alleviates T2DM through broader gut microbiota-mediated mechanisms compared to metformin, addressing both glucose and lipid dysregulation. Clinically, CCM offers a promising strategy for integrating TCM with modern medicine by targeting the gut-liver axis (38) and providing a systemic, holistic treatment approach. Moreover, the identification of specific probiotic candidates and key microbial pathways supports the development of precision medicine strategies rooted in TCM principles. These findings highlight CCM’s potential as a personalized and evidence-based therapy for T2DM, bridging traditional and modern medical approaches.

Our study has several limitations. First, the use of 16S rDNA gene sequencing lacks the resolution to distinguish microbial taxa at the species or strain level (53). This limitation hinders the identification and isolation of specific probiotic species or strains that may play pivotal roles in CCM’s therapeutic effects, further constraining the design and implementation of microbiota-based therapeutic validation experiments, such as microbial transplantation or supplementation. Second, while this study established correlations between enriched microbial taxa, their metabolic functions, and improved T2DM parameters, it did not fully explore the underlying therapeutic mechanisms driving these associations. Building upon these findings, future research should focus on leveraging advanced methodologies, such as metagenomics, metabolomics, and host transcriptomics, to uncover the molecular pathways and microbial–host interactions mediating CCM’s effects. These approaches would enable a more comprehensive understanding of the mechanisms underlying CCM’s therapeutic benefits and provide a foundation for developing targeted probiotic or microbiota-based therapies.

## MATERIALS AND METHODS

### Drugs and animal model

In our study, we utilized the CCM herbal formula, a TCM blend comprising three herbs: *Coptis rhizoma, Cinnamomi cortex*, *and Mume fructus*. These herbs were purchased from Shennong herbal slices Co., Ltd., located in Hebei Province, China, and were processed into boil-free granules (1). For the preparation of the CCM herb, each herb slice was accurately weighed according to the specified ratio. The preparation process involved adding water at 10 times the weight of the herbal mixture, followed by a 1 hour soaking period. The mixture underwent two extraction cycles, each lasting one hour. The extracts from both cycles were then combined and concentrated under reduced pressure at a temperature of 60°C, resulting in a concentrated decoction, with each milliliter equivalent to 31 g of dried medicinal herb. Metformin, catalog number B25331, was sourced from Yuanye Biotechnology Company, Shanghai, China. We used C57BL/KsJ(BKS) db/dbdiabetic mice and normal C57BL/Ks mice, both 8 weeks old, obtained from the Weitonglihua Corporation (Beijing, China).

### Study design and animal experiments

We obtained50 male db/db mice (BKS background) and 13 healthy male C57BL/Ks mice, all 8 weeks old, which were housed under standard laboratory conditions. The environment was maintained at a temperature of 22 ± 2°C, relative humidity between 40 and 60%, and a 12 hour light/dark cycle. After a 1-week acclimatization period, the db/db mice were randomly divided into four treatment groups: (i) the Low group (*n* = 9), which received a low dose of the CCM formula (2.3 g/kg/day); (ii) the Mid group (*n* = 11), which received a medium dose of the CCM formula (4.6 g/kg/day); (iii) the High group (*n* = 8), which received a high dose of the CCM formula (9.2 g/kg/day); and (iv) the MET group (*n* = 9), which was treated with metformin (0.3 g/kg/day). All treatments were administered via oral gavage once daily for a duration of 4 weeks. Additionally, the T2DM group (*n* = 13), consisting of db/db mice, did not receive any drug treatment during the same period. Body weight was monitored and recorded for each mouse throughout the study.

### Blood/Serum biochemical analysis

Blood samples were drawn from the tail vein of mice and stored at −80°C for subsequent analysis. FBG levels were measured using a Bayer Contour TS glucometer (Bayer, Hamburg, Germany). Serum levels of AST, ALT, CHOL, TGs, HDL-C, LDL-C, BUN, and CREA were determined using an automatic biochemical analyzer (LABOSPECT 003, Hitachi High-Technologies Co., Tokyo, Japan), in accordance with the manufacturer’s guidelines.

### Oral glucose tolerance test

Before the glucose tolerance test, mice were fasted for 12 hours. Baseline glucose levels were determined from a blood sample taken from the tail vein, using a Bayer Contour TS glucometer. A glucose solution (2 g/kg body weight) was prepared and administered orally. Glucose levels were then measured at intervals of 30, 60, and 120 minutes post-administration. These blood samples, taken from the tail vein, were analyzed using a glucometer. The resulting glucose measurements were plotted over time to calculate the AUC, as described by Sakaguchi et al. (54).

### Histopathological observation

Liver tissue sections were fixed in 4% paraformaldehyde for 24 hours and subsequently dehydrated through a graded series of ethanol concentrations followed by infiltration with dimethylbenzene. After embedding the samples in paraffin, 5 µm thick sections were obtained. The sections were then stained with hematoxylin and eosin. Histological images were captured using a digital camera system attached to a Leica microscope (Leica, Germany).

### Fecal DNA extraction

Fecal samples were collected from each mouse during the study and stored at −80°C. Genomic DNA was extracted from each fecal sample using a QIAmp DNA Stool Mini Kit (Qiagen, Valencia, CA, USA), following the manufacturer’s protocol. The quality of the extracted DNA was confirmed through 1% agarose gel electrophoresis.

### V4 16s rRNA gene region amplification and sequencing

Genomic DNA extracted from samples served as the template for amplifying the V4 region of the 16S rRNA genes. The PCR reactions, sequencing of PCR amplicons, and raw data quality control were conducted as previously described (2). Sequencing was carried out on an Illumina HiSeq 2500 platform (San Diego, CA, USA), producing 250 bp paired-end reads. These paired-end reads were merged using FLASH software (55). The high-throughput sequencing data of 16S rRNA gene amplicon reads (raw data) have been deposited in the National Genomics Data Center (GSA accession: CRA011391, https://bigd.big.ac.cn/gsa/browse/CRA011391). We employed QIIME2 version 2020.2 (56) to filter raw reads according to specific standards for high-quality read acquisition.

### Sequencing data analysis

Sequence analysis was conducted using VSEARCH version 2.0.3 (57). OTUs were generated from sequences with 97% or higher similarity. Representative sequences from each OTU were selected for further annotation. OTUs were classified taxonomically based on Silva_16s_v123 (58). OTU abundance data were normalized to the standard sequence count of the sample with the fewest sequences. Alpha- and β-diversity analyses were performed using QIIME2. For α-diversity, Richness and Shannon indices were calculated to assess community richness and diversity, respectively. Beta-diversity was analyzed by generating PCoA plots using Jaccard distance.

### Statistical analysis

To assess the statistical significance of clinical parameters across different groups, multivariate analysis was conducted using the R package ggpubr. Differential analysis of OTUs between groups was performed employing three independent methods: edgeR, STAMP, and LEfSe (25, 28, 29). Microbiota networks were constructed based on the relative abundance of OTUs in each group using the cluster_fast_greedy method, implemented in the R package ggClusterNet (27). The topological roles of OTUs in these networks were analyzed based on their within-module connectivity (Zi) and among-module connectivity (Pi) (26). To explore the functional coexistence of OTUs across different groups, we built networks for all differential OTUs based on robust correlations, using strong Spearman’s correlation coefficients (|*r*| > 0.8) and *P*-values less than 0.05.

Functional capacities of microbial communities were predicted using PICRUSt2 based on 16S rDNA sequences, and metabolic pathways were annotated according to the KEGG and MetaCYC databases (30). For identifying significant associations between microbial and phenotypic variables, we applied a linear multivariate regression model adapted for microbiome data (MaAsLin, Multivariate microbial Association by Linear models) (22). The related code is available on GitHub https://github.com/Jackwang87/XFF-herb-article-related-code.

## SUPPLEMENTAL MATERIAL

Supplemental figures spectrum.02412-24-s0001.docx Figures S1 to S7.

Table S1 spectrum.02412-24-s0002.xlsx Differential operational taxonomic units (OTUs) between the T2DM group and the treatment groups (MET/Low/Mid/High-wk4) analyzed by edgeR, STAMP, and Lefse.

ASM does not own the copyrights to Supplemental Material that may be linked to, or accessed through, an article. The authors have granted ASM a non-exclusive, world-wide license to publish the Supplemental Material files. Please contact the corresponding author directly for reuse.
