# Supplementary material for: Gut microbiota’s role in the enhancement of type 2 diabetes treatment by a traditional Chinese herbal formula compared to metformin
Source: Microbiol Spectr. 2025 Mar 31;13(5):e02412-24. doi: 10.1128/spectrum.02412-24 (PMC12054121; doi:10.1128/spectrum.02412-24)
Supplement: Supplemental figures — Figures S1 to S7. [file spectrum.02412-24-s0001.docx]

**Gut microbiota’s role in the enhancement of type 2 diabetes treatment by a traditional Chinese herbal formula compared to metformin**

Chengdong Xia ^a, 1^, Liya Yue ^b, c, 1^, Yinyu Wang ^b, c, 1^, Cuidan Li ^b, c, 1^, Guannan Ma ^b, c, 1^, Yingjiao Ju ^b, c, d^, Peihan Wang ^b, c, d^, Jie Wang ^b, c, d^, Xiaoyuan Jiang ^b, c^ Xiaotong Wang ^b, c, **^, and Fei Chen ^b, c, d, e, f, g, *^

^a^ Department of Endocrinology, Xiyuan Hospital of China Academy of Chinese Medical Sciences, Beijing 100091, China

^b^ China National Center for Bioinformation, Beijing 100101, China

^c^ Beijing Institute of Genomics, Chinese Academy of Sciences, Beijing 100101, China

^d^ University of Chinese Academy of Sciences, Beijing 100049, China

^e^ State Key Laboratory of Pathogenesis, Prevention and Treatment of High Incidence Diseases in Central Asia, Clinical Medicine Institute, The First Affiliated Hospital of Xinjiang Medical University, Urumqi 475000, China

^f^ Key Laboratory of Viral Pathogenesis & Infection Prevention and Control (Jinan University), Ministry of Education, Guangzhou 510632, China

^g^ Beijing Key Laboratory of Genome and Precision Medicine Technologies, Beijing 100101, China


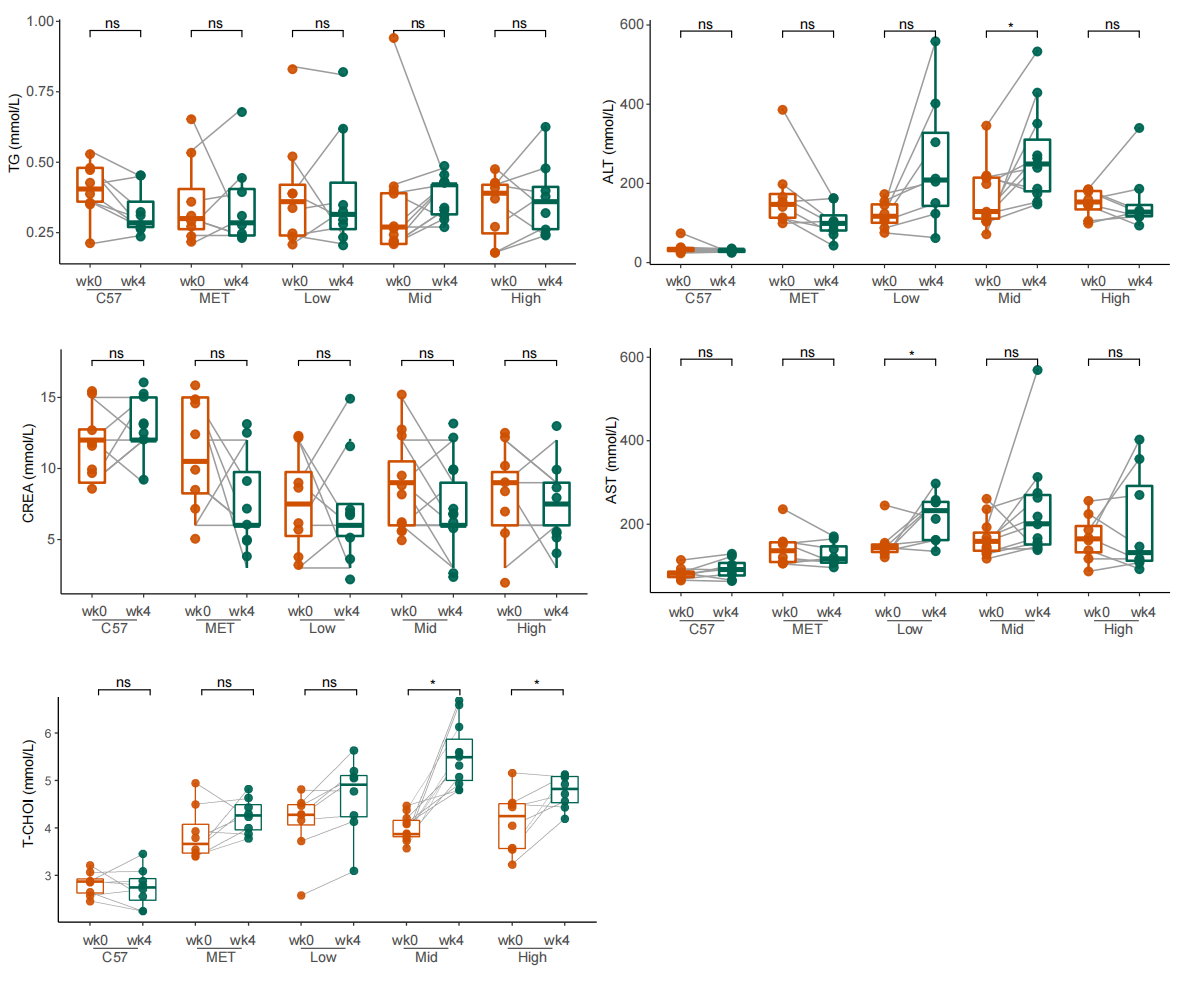


**Fig. S1** The Effects of XFF and Metformin treatment on triglyceride (TG), aminotransferase (AST), alanine aminotransferase (ALT), creatinine (CREA) and total cholesterol (T-CHOI).


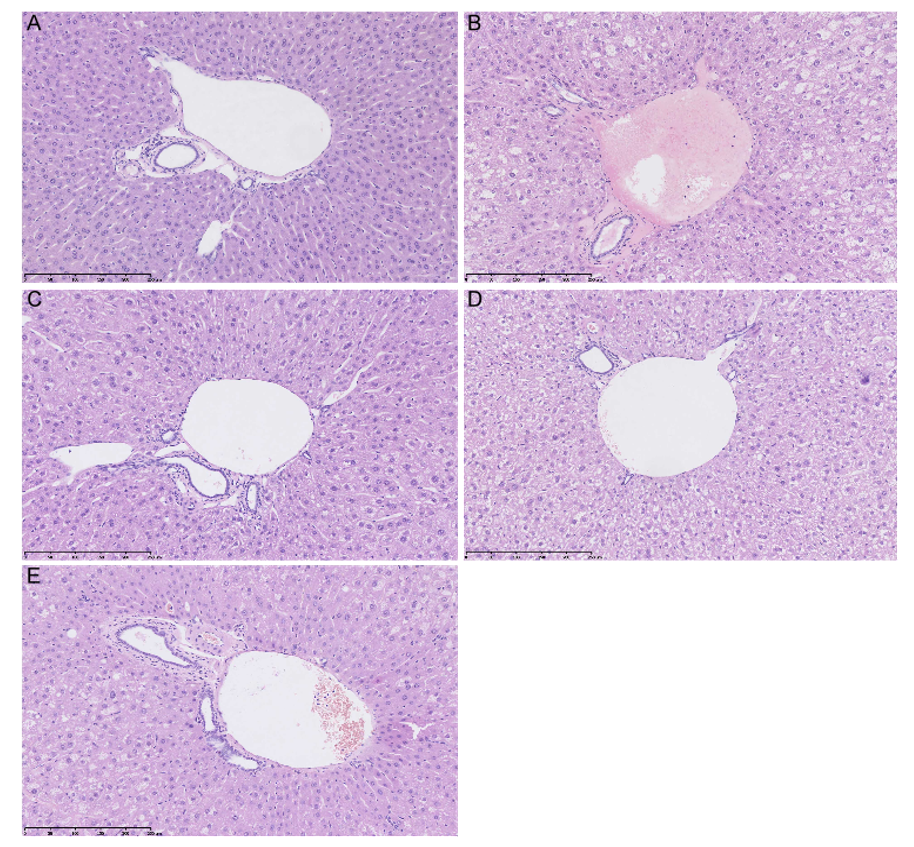


**Fig. S2** Liver section of a mouse stained with HE, showing the portal area. (A) Normal mice: No fat accumulation, normal hepatocyte arrangement. (B) Diabetic mice: Significant fat accumulation, hepatocytes appear vacuolated with notable fat droplets. (C) Metformin treatment: Fat accumulation is reduced, but mild fat degeneration persists, showing some improvement in liver condition. (D) Low dose of CCM treatment: Fat accumulation decreases, and fat degeneration is alleviated. (E) High dose of CCM treatment: Fat accumulation is significantly reduced, hepatocyte morphology is closer to normal, and fat degeneration is greatly diminished. Scale bars = 250 µm.


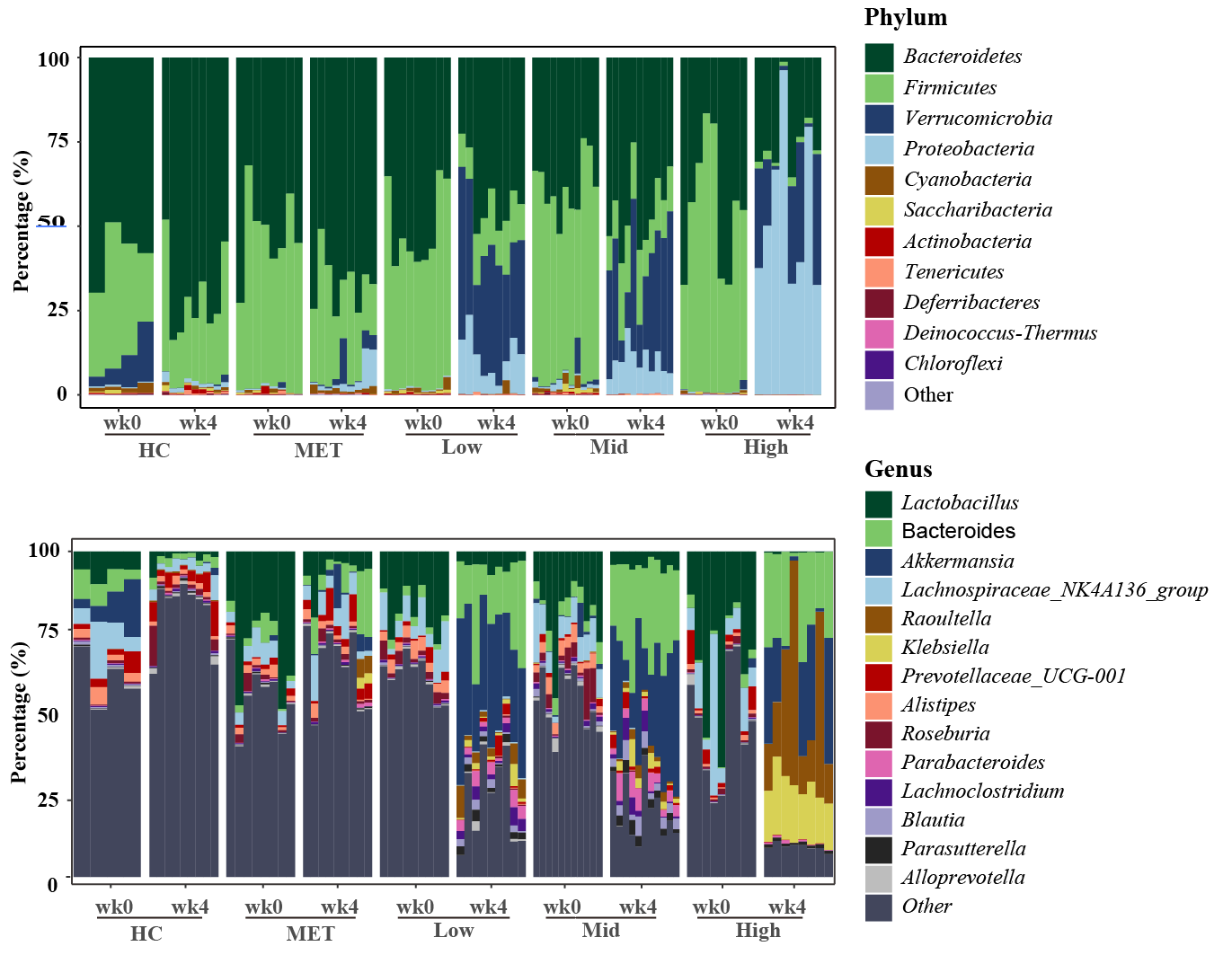


**Fig. S3** Taxonomic composition of the gut microbiota in samples treated with different doses of XFF formula and Metformin at weeks 0 and 4 using normal mice as healthy control.


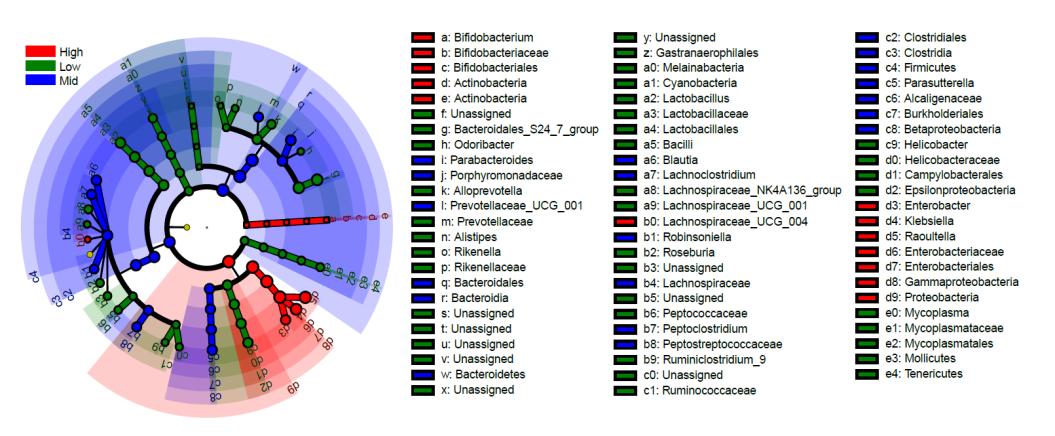


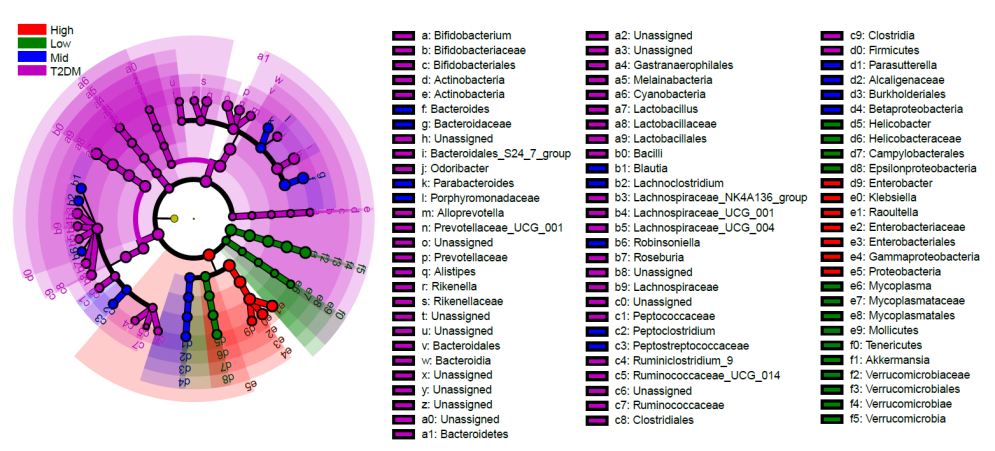


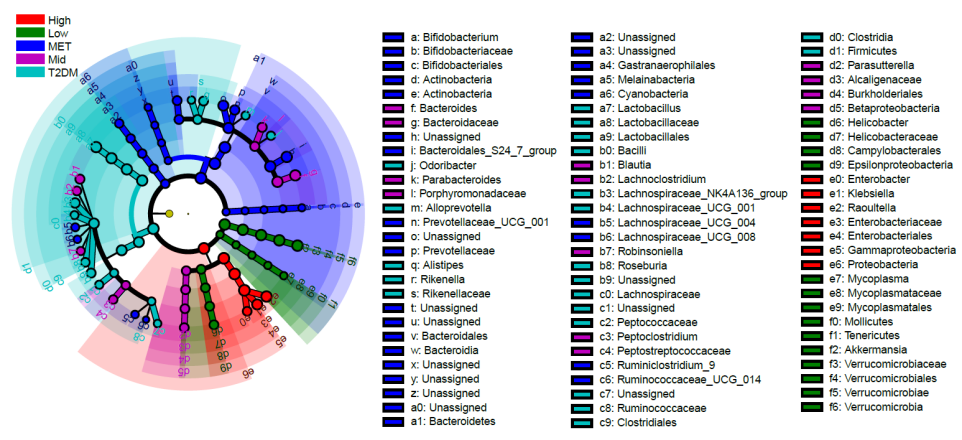


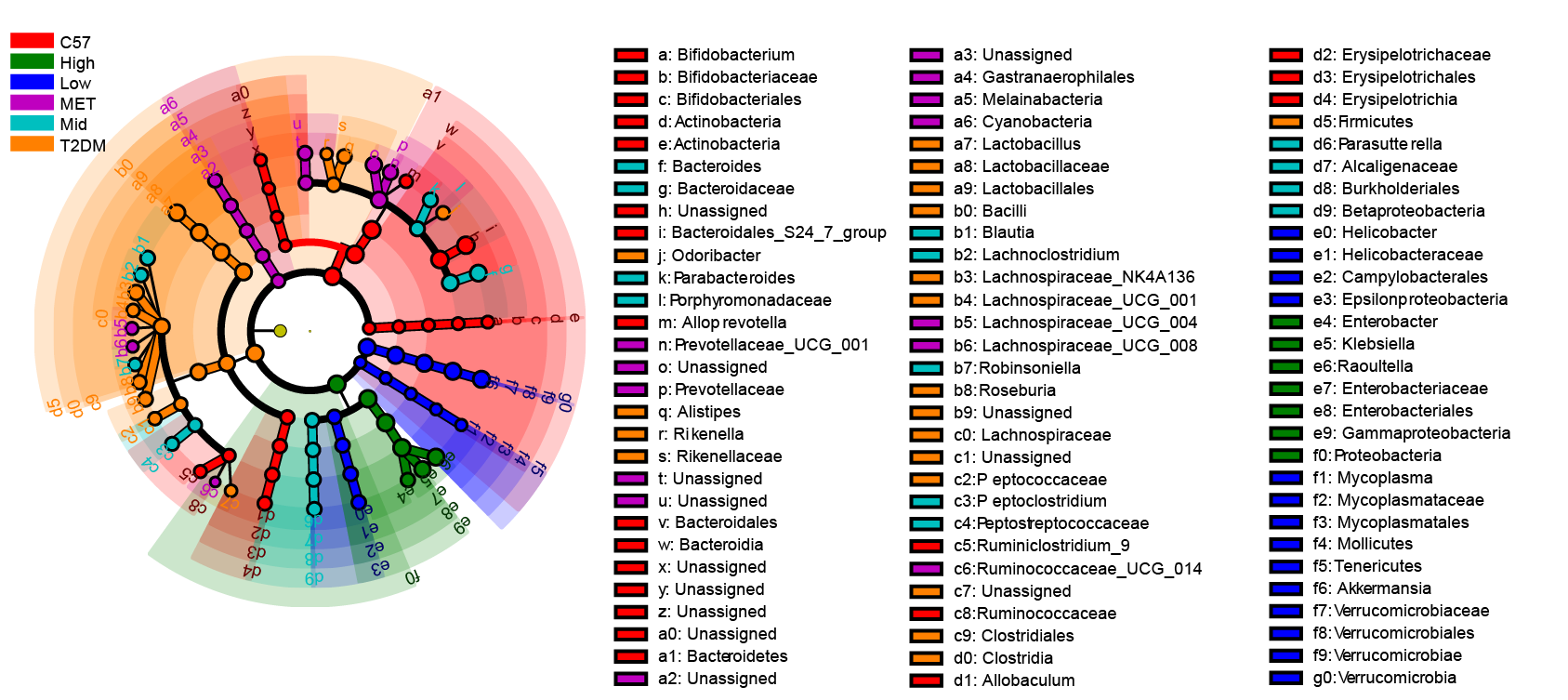


**Fig. S4** LEfSe identification of the significantly differential bacterial taxa between different comparison groups.


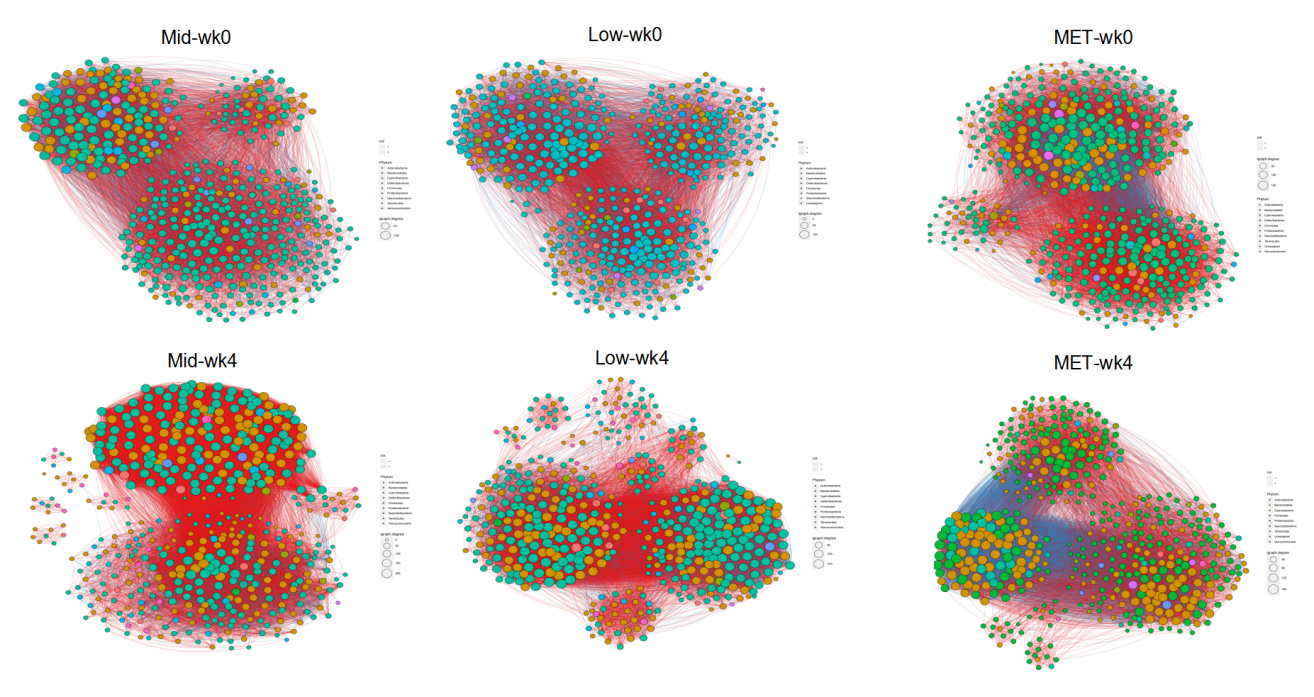


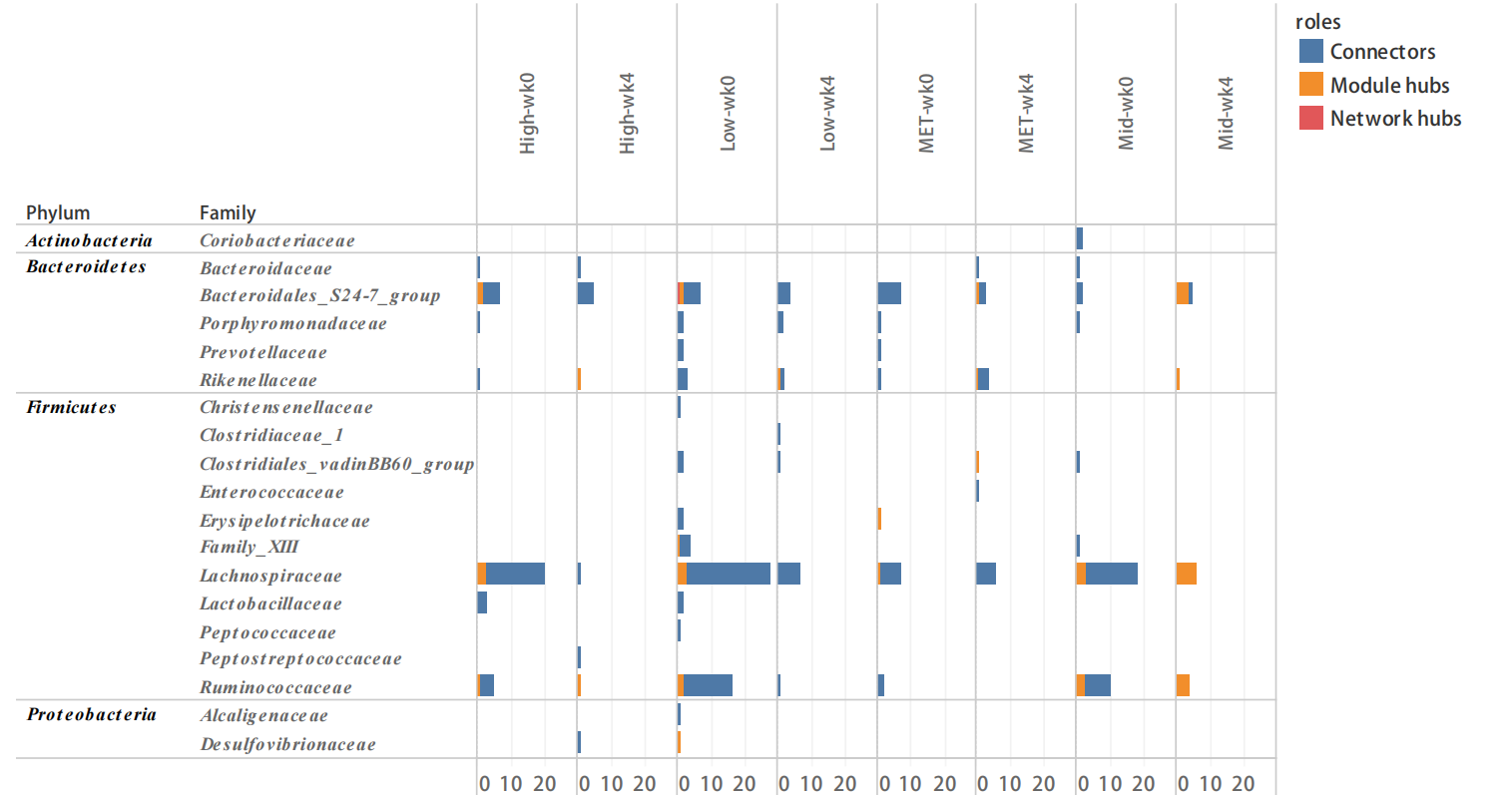


**Fig. S5** Co-abundance networks of the gut microbiota in T2DM mice treated with metformin (MET) and Low/Mid dose XFF groups at weeks 0 and 4. The numbers of OTUs with special topological roles are assigned to different taxon in the networks and summarized in the table.

**Fig. S6** (A)Taxonomic profiles of the OTUs in different modules (B) Key OTUs in the modules significantly associated with the T2DM clinical features. Nominal P values and FDR corrected q-values are assigned by MaAsLin2.

**Fig. S7** The bacterial metabolic pathways identified closely associated with and body weight, spartate aminotransferase (AST), alanine aminotransferase (ALT).
